# Implementation Approaches for Introducing and Overcoming Barriers to Hepatitis B Birth-Dose Vaccine in sub-Saharan Africa

**DOI:** 10.9745/GHSP-D-21-00277

**Published:** 2022-02-28

**Authors:** Alix Boisson, Varun Goel, Marcel Yotebieng, Jonathan B. Parr, Bruce Fried, Peyton Thompson

**Affiliations:** a Department of Health Policy and Management, Gillings School of Global Public Health, University of North Carolina at Chapel Hill, Chapel Hill, NC, USA.; b Department of Geography, University of North Carolina at Chapel Hill, Chapel Hill, NC, USA; Carolina Population Center, Chapel Hill, NC, USA.; c Division of General Internal Medicine, Department of Medicine, Albert Einstein College of Medicine, Bronx, NY, USA.; d Division of Infectious Diseases, Department of Medicine, University of North Carolina School of Medicine, Chapel Hill, NC, USA.; e Department of Health Policy and Management, Gillings School of Global Public Health, University of North Carolina at Chapel Hill, Chapel Hill, NC, USA.; f Division of Infectious Diseases, Department of Pediatrics, University of North Carolina School of Medicine, Chapel Hill, NC, USA.

## Abstract

In sub-Saharan Africa (SSA), chronic viral hepatitis B (HBV) affects more than 60 million people. Mother-to-child transmission is a major contributor to the ongoing HBV epidemic and yet only 11 of 54 (20.3%) SSA countries have introduced the birth dose of HBV vaccine (HepB-BD) into their regular immunization schedule. As more African countries adopt HepB-BD, implementation approaches must be targeted to ensure effective and timely HepB-BD delivery, especially in rural and under-resourced settings. We conducted a systematic literature review of published literature using PubMed. We included 39 articles published from January 2010 to August 2020, as well as gray literature, case studies, and research performed in SSA. We describe barriers to the uptake of HepB-BD in SSA at the policy, facility, and community levels and propose solutions that are relevant to stakeholders wishing to introduce HepB-BD. We highlight the importance and challenge of reaching infants who are born outside of health care facilities (i.e., home deliveries) with HepB-BD in partnership with community health workers. We also discuss the critical role of maternal education and community engagement in future HepB-BD scale-up efforts in SSA.

## Abstract (precis)

We discuss determinants of hepatitis B birth-dose vaccine uptake in sub-Saharan Africa countries at the policy, facility, and community levels and propose solutions to known barriers of hepatitis B vaccine introduction in low- and middle-income countries.

## Teaser

**Key Findings:**

- A review of 39 articles outlining the experience of introducing the hepatitis B birth-dose (HepB-BD) vaccine in low- and middle-income countries in sub-Saharan Africa identifies the potential barriers to the vaccine's uptake.
- Empirical examples of policy makers targeting the mother's role to increase uptake of HepB-BD vaccine at the community level are strikingly scarce, and efforts to leverage community-level resources and reach remote areas have been limited.

**Key Implications:**

- We provide solutions that must be adapted to the local context to overcome the barriers to HepB-BD uptake identified across policy, facility, and community levels.
- Synergy must exist between policy, facility, and community level implementation for sub-Saharan African countries to effectively implement a universal HepB-BD vaccine immunization policy.
- For sub-Saharan African country policy makers to effectively implement a universal HepB-BD immunization policy, synergy must exist between the policy-, facility-, and community-level efforts.

## INTRODUCTION

Six years after the World Health Organization (WHO) African Regional Committee convened to develop a hepatitis elimination strategy, hepatitis B virus (HBV)-related mortality remains high at 200,000 deaths per year in sub-Saharan Africa (SSA).^1^^,^^2^ The committee identified the prevention of HBV in children as a priority, aspiring to lower seroprevalence of hepatitis B surface antigen-positivity among children aged younger than 5 years to less than 2% by 2020 and less than 0.1% by 2030.^3^

Mother-to-child transmission (MTCT) is a significant driver of the ongoing HBV epidemic. HBV exposure at a young age is associated with more severe liver disease; if infected at birth, an infant has a 70%–90% chance of developing chronic HBV.^4^^,^^5^ The WHO estimates that chronic HBV affects more than 100 million individuals in the African region.^4^ It has been estimated that over 350,000 infants are infected annually via MTCT in the African region.^6^ Thus, preventing MTCT of HBV is a cornerstone of effective strategies against the HBV epidemic in SSA. The birth dose of HBV vaccine (HepB-BD) is the most effective (>95%) prevention measure against MTCT if administered within 24 hours of birth^7^^,^^8^ and followed by completion of 2 or 3 routine immunizations.^9^ Despite the availability of the HBV vaccine since 1982, few SSA countries administer the birth-dose vaccination due to implementation challenges and a lack of HBV burden awareness.^10^

Despite the availability of the HBV vaccine since 1982, few SSA countries administer the birth-dose vaccination due to implementation challenges and a lack of HBV burden awareness.

The global health community understands the importance of the HepB-BD vaccine, which has led to Gavi, the Vaccine Alliance sponsoring distribution costs for the HepB-BD vaccine across SSA.^11^^,^^12^ Though these efforts are ongoing, as of April 2021, only 13 of 48 countries (27%) in SSA have introduced HepB-BD,^13^ falling short of the WHO Africa Regional Committee goal of at least 25 countries by the end of 2020^3^ (Figure).^13^^–^^14^ A majority of SSA countries still use the standard HBV vaccine schedules (3 doses of hepatitis B vaccine), typically given at 6, 10, and 14 weeks of life. Although this schedule is practical, immunizing infants beginning at 6 weeks does not prevent vertical transmission from HBV-infected mothers to their infants.

**FIGURE:**
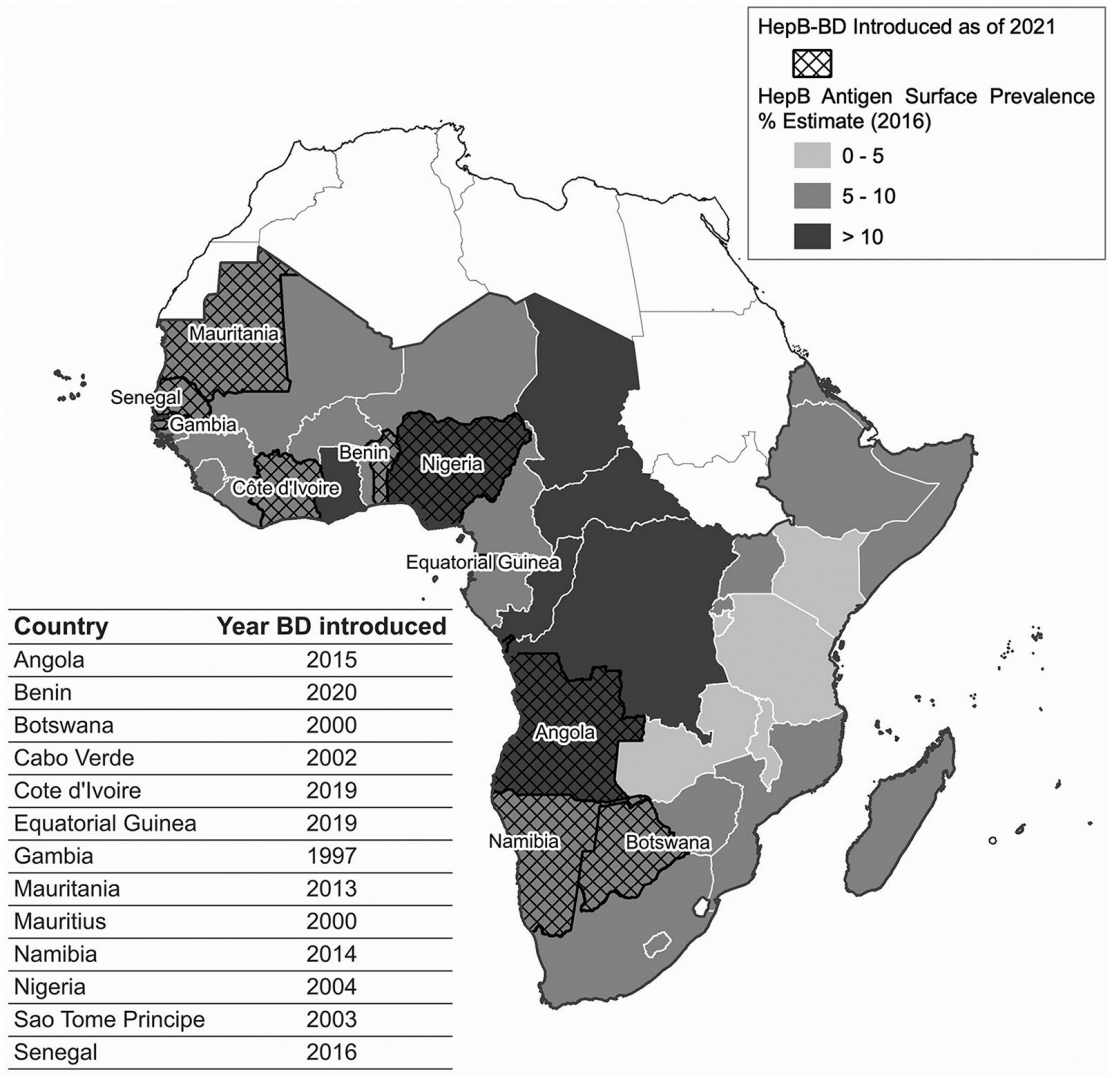
Choropleth Map Demonstrating Hepatitis B Birth-Dose Vaccine Coverage and Hepatitis B Surface Antigen Prevalence in sub-Saharan Africa^a^ Abbreviations: HepB-BD, hepatitis B birth dose; Hep B, hepatitis B; BD, birth dose. ^a^ This figure presents the countries in sub-Saharan Africa (SSA) that have introduced the birth dose of HBV vaccine (HepB-BD) and estimates of HepB antigen surface prevalence by country. SSA islands not depicted in the map include Cabo Verde, Comoros, Mauritania, Mauritius, São Tomé and Principe, and Seychelles. The table lists the year of HepB-BD introduction for all SSA countries offering the vaccine by 2021. All classification intervals are left closed and right open. Geographical boundaries obtained from Global Administrative Areas version 3.6. Source: Razavi-Shearer et al.; Njuguna.

Barriers to HepB-BD implementation in SSA exist across the supply chain and care continuum. Key barriers include lack of evidence of HBV seroprevalence and rates of MTCT, financial costs of vaccine distribution, insufficient cold-chain storage, lack of trained community health workers (CHWs), and a high proportion of at-home births.^4^ Given these challenges, HepB-BD implementation practices must be in place alongside vaccine distribution to ensure effective vaccine delivery, especially in rural and under-resourced settings. We discuss existing determinants of HepB-BD uptake in SSA countries at the policy, facility, and community levels and propose solutions for stakeholders to introduce HepB-BD in low- and middle-income countries (LMICs). While the literature often discusses solutions at the policy and facility levels, we argue that researchers and stakeholders should place more emphasis on community-level interventions, especially in highly rural contexts.

## METHODS

Iterative scoping reviews were performed using PubMed to identify articles published between January 2010 and August 2020 that discussed methods for country-level implementation of HepB-BD. We used the following key terms in the search: “hepatitis B birthdose vaccination,” “HepB-BD,” “hepatitis B birth dose vaccination,” “hep b birthdose,” and “HBV birth dose vaccination.” Because immunization system implementation science is relevant to HepB-BD implementation efforts in LMIC settings, we also reference this broader swath of the scientific literature. Literature was drawn only from articles published after 2010 to maintain relevance, as evidence-based immunization implementation science is a fast-developing field. In addition to articles identified through PubMed, we also extracted findings from gray literature, case studies, and research performed in SSA, but we expanded the search to Southeast Asia (SEA), in part due to the successful introduction of HepB-BD in SEA.

## RESULTS

In all, 598 reports were identified, and 39 were relevant and reviewed for this publication (Table 1 and Supplement). To be included, articles had to describe research introducing the HepB-BD within an LMIC or data-backed guidance for the inclusion of HepB-BD. Of articles included, 13 reports focused solely on SSA. We categorize vaccine uptake determinants in 3 domains with immediate relevance to HepB-BD implementation: policy-, facility-, and community-level barriers and solutions. Major themes, barriers, and solutions are outlined in Table 2.^15^^–^^32^

**TABLE 1.** Hepatitis B Birth Dose Vaccine Uptake Themes Discussed in the Empirical Literature (N=39)

	**Sub-Saharan Africa Specific**	**Policy-Level**	**Facility-Level**	**Community-Level**
Breakwell et al., 2017^4^	X	X	X	X
Reardon et al., 2019^9^	X	X	X	
Spearmen et al., 2017^10^	X		X	X
Dionne-Odom et al., 2018^11^	X	X	X	X
Nelson et al., 2016^12^		X	X	X
Boa et al., 2017^15^	X	X		
Chang et al., 2019^16^			X	
Ginzberg et al., 2018^17^		X	X	
Hambridge et al., 2019^18^	X	X		
Howell et al., 2014. ^19^	X	X	X	
Jourdain et al., 2019^20^		X	X	
Kolwaite et al., 2016^21^				X
Mak et al., 2018^22^				
Miyahara et al., 2016^23^	X		X	X
Nayagam et al., 2016^24^		X	X	
Nayagam et al., 2016^25^		X	X	
Nguyen et al., 2019^26^				X
Pham et al., 2018^27^			X	
Scott et al., 2018^28^		X		
Sobel et al., 2011^29^		X	X	
Spearman, 2018^30^		X		X
Wiesen, et al., 2016^31^		X		
World Health Organization, 2019^32^		X	X	X
Okenwa et al., 2019^33^	X	X	X	X
Breakwell et al., 2017^34^			X	X
Moturi et al., 2018^35^	X		X	X
Awuku and Yeboah-Afihene, 2018^36^	X	X	X	
Tamandjou et al., 2017^37^	X	X	X	X
Hagan et al., 2019^39^		X	X	
Anderson et al., 2018^40^	X	X	X	
Beigi et al., 2014^47^				X
Li et al., 2017^52^			X	X
Lemoine, Thursz, 2017^56^	X	X		
Centers for Disease Control and Prevention, 2013^57^			X	X
Pham et al., 2019^59^				X
Giao et al., 2019^60^				X
Woodring et al., 2019^62^			X	X
Xeuatvongsa et al., 2016^64^				X
Wiesen et al., 2016^68^			X	X

**TABLE 2:** Potential Barriers to and Solutions to Hepatitis B Birth-Dose Vaccine Uptake in Sub-Saharan Africa Countries

**Intervention Level**	**Determinant**	**Potential Barriers**	**Potential Solutions**
Policy	Lack of political willingness: advocacy	Lack of awareness of importance of vaccine	Engage relevant stakeholders, decision makers, and effective in-country advocacy groups
		Lack of vaccine advocacy	Establish national-level policy mandates for the timely delivery of HepB-BD
	Lack of political willingness: affordability	Lack of available resources	Provide cost-effective examples in sub-Saharan Africa
		Lack of awareness of quantitative impact of vaccine	Draw on drug manufacturer or other donors for distribution cost support
	Need for effective recommendations	Lack of consensus recommendations for vaccine implementation	Develop site-specific recommendations that draw upon research and literature, international guidelines, and feedback from diverse stakeholders
Facility and Logistics	Knowledge and training of health workers	Lack of awareness of vaccine benefits, stigma, and gaps in knowledge among CHWs	Educate facility staff on the HepB-BD vaccine and administration protocol
			Cultivate champions
			Comprehensive training
			Completion of checklist form by staff before discharge of newborn
			Couple immunization with BCG and oral polio vaccine
		Variable vial size and concern for wastage	Make available vial size combinations
	Short window for administering vaccine	24-hour administration window	Keep mothers in delivery ward at least 24 hours after delivery
		Mother's hesitancy to vaccinate infant	Administer vaccine in delivery ward
			Mitigate cost burden
	Cost burden	User fees for vaccines	Subsidize or reduce costs associated with regular immunization
	Tracking systems	Lack of adequate reporting infrastructure	Standardize all Hep B-BD immunization-reporting tools
		Faltering vaccine recording buy-in by facility staff	
	Vaccine storage and stockouts	Limited storage space and stock-out determinants	Store the vaccine in existing cold chains and/or in labor wards
			Allow private providers to obtain the vaccine free-of-charge
		Poor communication between the immunization and maternity wards	Establish standing orders for the vaccine
		Reaching remote rural villages with vaccines	Cultivate partnerships with vaccine distributor
Community	Maternal involvement	At-home births	Leverage post-home birth visit to administer vaccine
			Raise vaccine awareness within the community
		Geographic distance inhibiting timely delivery of newborn to health facility	Educate mothers during antenatal care visits
			Families to keep home-based records
	Community health worker involvement	Poor communication channels between CHWs and mothers	Perform home visits in rural communities to educate mothers, track pregnancies, and refer mother-infant pair to nearby facilities
			Provide at-home immunization for infants
			Strengthen ties between CHWs and facilities
			Engage community leaders and members
			Provide incentives for CHWs
	Evidence-based innovations to reach communities	Vaccine refrigeration requirements	Use of out-of-cold-chain or controlled temperature chains
			Mobile-based devices to track pregnancies in rural areas

Abbreviations: BCG, Bacillus Calmette-Guerin; CHW, community health worker; HepB-BD, hepatitis B birth dose.

### Policy Level

The first step toward HepB-BD implementation requires the development of clear policy recommendations and changing national vaccine schedules to include HepB-BD. Advocates and other stakeholders must first synthesize setting-specific evidence of HBV prevalence and solutions. Next, policy makers and other relevant players must be engaged to convey the need for HepB-BD and relevant policy change. These efforts must address 2 persistent barriers to national policy change: a lack of political will and insufficient evidence.

Advocates, policy makers, and other stakeholders working to change national vaccine policy must address the barriers of lack of political will and insufficient evidence.

#### Lack of Political Willingness: Advocacy

Countries often lack the political will to enact policies that mandate HepB-BD within 24 hours of birth, ensure vaccine availability at every facility, and ensure an adequate supply chain of HepB-BD.^33^ A lack of awareness of the vaccine’s effectiveness and insufficient advocacy for HepB-BD often contribute to the policy-level determinants. Vaccine supporters can foster political will by engaging relevant stakeholders, decision makers, and effective in-country advocacy groups across all health care systems and socioecological levels.

Engaging a united interdisciplinary group of stakeholders is more effective for supporting multilevel HepB-BD implementation efforts to address existing barriers. Primary stakeholders may include ministries of health and finance, donor agencies, external academic and nongovernmental or multilateral organizations involved in the programming, and drug manufacturers. Relevant literature also stresses the importance of other groups in achieving political commitment for in-country HepB-BD uptake, such as professional societies, medical associations, and community and religious leaders.^34^

São Tomé and Príncipe and Nigeria demonstrated success by calling on the private health sector, physicians and nurses, and hepatology associations in advocacy efforts to steer the government towards introducing HepB-BD.^35^^,^^36^ Although stakeholder buy-in is crucial in any setting, country-specific groups and associations differ by context, and setting-specific partners should be identified and supported. This collaborative approach is required to develop an effective and sustainable intervention with HepB-BD champions at every socioecological level. To achieve uptake, stakeholders should understand the importance of advocating alongside partners in different but related sectors such as cancer prevention disease-specific advocacy groups^4^^,^^37^ or safe motherhood care. Strengthening the case for universal HepB-BD by engaging diverse stakeholders will press decision makers to pledge political commitment.

Finally, advocacy among decision makers for national policies to mandate the timely delivery of the vaccine within the 24-hour requirement and the pre-positioning of vaccines in the maternity wards are both critical to ensuring high uptake of HepB-BD. A study in Nigeria provided recommendations for improving HepB-BD uptake among infants.^33^ During the time of the study, national-level policy makers in Nigeria had not introduced a policy mandate. While the National Primary Health Care Development Agency in Nigeria recommends that all infants receive HepB-BD, the immunization window ranges from the birth of the newborn to 2 weeks of age. One of the study’s primary recommendations was to mandate a policy for HepB-BD immunization of newborns within 24 hours of delivery. Compared to a similar study in the Philippines that had introduced a policy mandating infant immunization within the 24-hour window, the uptake of HepB-BD in Nigerian hospitals (26.2%) was much lower than in the Philippines hospitals (87%).^33^

Advocacy among decision makers for national policies to mandate the delivery of HepB-BD within the 24-hour requirement and the pre-positioning of vaccines in the maternity wards are both critical to ensuring high uptake of the vaccine.

#### Lack of Political Willingness: Affordability

In addition to advocacy, affordability and cost-effectiveness are key determinants of political willingness. Deceleration of vaccine uptake occurs due to financial uncertainty or concern over the availability of resources.^37^ Quantitative impact estimates are recommended for policy makers, as they solidify plans for start-up, maintenance, and opportunity costs. Cost-effectiveness and scalability analyses can be achieved using models derived from country-specific pilot studies or similar studies from neighboring countries.

Experience from other SSA countries can be used to demonstrate a favorable return on investment. Preliminary research confirms the affordability of HepB-BD, which costs only US$0.20 per 10-dose vial, making it one of the least expensive vaccines available.^11^^,^^34^^,^^36^^,^^38^ Various studies analyze the affordability of HepB-BD both in SSA and in SEA.^9^^,^^39^ A notable study comparing 3 strategies of universal HepB-BD, targeted HepB-BD, and the pentavalent vaccine only indicated that the addition of a universal HepB-BD prevented the largest number of additional MTCT cases and was the preferred strategy at a willingness-to-pay threshold of US$150 per infection prevented.^40^ Success stories from other SSA countries are also useful in achieving political commitment because they demonstrate clear return-on-investment through sustained improvements in coverage rates following policy change. Empirical successes include the introduction of HepB-BD into the Gambia in the 1990s, Botswana in the 1990s, Namibia in 2014–2015, and São Tomé and Príncipe in 2019.^35^^,^^39^

The vaccine’s scalability improves returns^9^^,^^39^ but also introduces challenges in building adequate vaccine reserves and meeting costs of large-scale distribution. A solution is to apply for sponsorship from Gavi,^11^^,^^12^ support that is especially important when a government lacks budgetary flexibility. During the coronavirus disease (COVID-19) pandemic, donors’ financial commitments have shifted from noncommunicable to infectious diseases, especially in LMICs. Countries around the world may turn to LMICs to guide how best to tackle infectious diseases. While global stakeholders are recognizing the risk of communicable diseases, maintaining regular vaccinations is now more critical than ever to avoid the emergence of twin epidemics. The time is right to secure funding for the roll-out of the HepB-BD **now** to pilot a successful vaccine distribution initiative that can serve as an example for future large-scale COVID-19 vaccine campaigns.

#### Need for Effective Recommendations

Consensus recommendations for HepB-BD implementation are lacking but necessary to translate political will into effective action. We suggest developing site-specific recommendations that draw upon research and literature, international guidelines,^41^ and feedback from diverse stakeholders, including people living with HBV.^42^ We propose 2 ingredients for building a strong recommendation: a well-constructed strategy based on local experience and the application of knowledge gleaned from implementation theory perspectives. It is crucial to develop a comprehensive implementation strategy from the outset that is grounded in theory and uses the latest innovations to overcome any barriers to HepB-BD uptake.^43^ Public health policies and practices disseminated by WHO are a logical starting point and are guided by the most comprehensive health research knowledge to improve global health worldwide.^44^^,^^45^ Additionally, research from neighboring and comparable settings should be used to develop recommendations.^41^ Finally, implementers should continuously revisit and evaluate HepB-BD recommendations to incorporate recent innovations and improve any implementation determinants.^46^

It is crucial to develop a comprehensive implementation strategy from the outset that is grounded in theory and uses the latest innovations to overcome any barriers to HepB-BD uptake.

### Facility Level and Logistics

The primary delivery method for HepB-BD is health facilities, which include public hospitals, clinics, health centers, and private health facilities. Given the diversity of settings where HepB-BD might be delivered, attention should be given to setting-specific challenges such as lack of community health workers’ (CHW) knowledge/training on HBV and prevention through vaccination, logistical challenges in administering the vaccine within 24-hours of birth, difficulty in tracking newborn vaccine status, vaccine stock-outs, systemic vaccine stigma, local opportunity costs, and the negative impact of user fees for immunizations.

#### Knowledge and Training of Health Workers

Provider-level HepB-BD knowledge in health facilities is an essential driver of vaccine uptake. Knowledge may be lacking at 2 levels: recognizing the benefits of HepB-BD and general training for timely administration of the vaccine.^35^^,^^47^ Lack of awareness of vaccine benefits, stigma, and gaps in knowledge among CHWs, as well as variable vial size and concern for wastage, may hinder HepB-BD uptake.^35^^,^^47^

Provider knowledge may be lacking at 2 levels: recognizing the benefits of HepB-BD and general training for timely administration of the vaccine.

One solution to address the lack of awareness and overcome stigma about HepB-BD is educating staff on the vaccine’s benefits and shortcomings. Sharing success stories demonstrating HepB-BD’s effectiveness can educate and motivate facility staff and cultivate HepB-BD champions.^47^ Data should be shared on the only reported negative consequence of HepB-BD—an anaphylactic reaction caused by previous HBV exposure—which rarely occurs after about 1 in 1 million doses of the vaccine.^35^ Alleviating any fear or bias against vaccinating newborns and ensuring provider buy-in is critical for introducing HepB-BD in their communities and ensuring its sustainability.

While HepB-BD’s narrow time window may reduce provider willingness to adopt the vaccine, providing straightforward written guidelines, conducting initial and refresher training, and providing supportive supervision can ease the burden of the vaccine’s specific logistical challenges.^35^ Training should occur in all facilities within a community—whether public or privately operated. The complete multidisciplinary workforce should be involved to ensure vaccine coverage for all involved in the training/vaccination process in anticipation of wide-scale distribution.^34^^,^^35^ Training objectives may include assigning vaccine-related duties to a specific role. A study in China determined the success of a strategy incorporating the vaccine-administration role to the obstetrician, clearing up potential confusion in terms of vaccination responsibilities.^34^^,^^35^ A Nigerian study found that vaccine oversights were vastly avoided when staff completed a vaccine-related checklist before discharging mother-infant pairs.^35^ Finally, staff may be trained to streamline the HepB-BD vaccine with the vaccines for TB and polio to improve timely vaccination. To date, 4 SSA-based studies have assessed the effect of all 3 vaccines outside of the 24-hour window, 5 studies have measured the rate of individual vaccine uptake within 24 hours of delivery, but no study has evaluated the effect of streamlining the 3 vaccines at birth.^48^ The WHO confirms that the 3 vaccines do not interfere with one another’s immune responses.^45^ Since the facility already offers one or both vaccines, health staff may leverage existing training experience for administering HepB-BD.^45^

Another challenge to administering the vaccine in the 24-hour window is the various vial sizes (single-, 2-, 6-, 10-, or 20-dose vials).^45^ In the case of a small number of deliveries within a given period, facility staff often wait to open multidose vials to avoid wastage. To address this issue, vaccine suppliers could consider making available a combination of single-dose and multidose vials, such that vial size would be appropriate for both smaller facilities with fewer daily deliveries and larger facilities with multiple daily deliveries. Facility staff should receive training on a protocol specific to their facility’s volume of deliveries and vial sizes. Some would argue that vials should be opened regardless of concerns for wastage because the benefits of timely vaccination far outweigh the risk of wastage.

#### Short Window for Administering Vaccine

The HepB-BD vaccine’s short window for administration is perhaps its greatest implementation challenge. Administering timely vaccine is difficult for births that occur in facilities. It is even more pronounced for infants born outside of facilities (see discussion of at-home births in the community-level section). Maternal hesitancy and lack of awareness of HBV and the HepB-BD may further complicate efforts to administer timely infant vaccination in facilities. A mother's hesitancy may be due to general or HepB-BD-specific vaccine hesitancy, cost, or other factors, but the short window for vaccine administration requires an effective approach to generating mother buy-in quickly.

The HepB-BD vaccine’s short window for administration is perhaps its greatest implementation challenge.

The WHO suggests keeping mothers in the delivery ward for at least 24 after their child’s birth, a practice not widely adopted in SSA health facilities.^35^ A study in São Tomé and Príncipe reported combining 2 approaches: for mother-infant pairs to remain in the postnatal ward for longer than 1 day and to offer vaccines in the maternity ward for on-site vaccination. This joint approach yielded improved accessibility, greater convenience, and increased HepB-BD uptake.^35^^,^^39^

#### Cost Burden

Cost is a critical barrier to consumer uptake (in addition to the previously discussed affordability consideration on a national level) that must be carefully addressed. User fees may prevent families from taking their newborns to receive vaccinations at higher-quality or preferred facilities. While vaccines in many countries are provided free-of-charge to facilities, most providers request a payment for a vaccination card to cover administrative and workforce costs for immunization services and monitoring during the infant’s first year of life.^49^

Removing monetary barriers through small cash incentives or reducing user fees for immunization can improve uptake in the short-term,^49^^,^^50^ though it may not be a sustainable long-term option. A study in the Democratic Republic of the Congo demonstrated that uptake of the rotavirus vaccine was 99% if the vaccine was provided free of charge.^51^ In the long term, policy makers may consider using national- or district-level budgets to subsidize administrative costs accrued by facilities to improve regular immunization rates among infants.

#### Tracking Systems

The current SSA practice for complete immunization following HepB-BD includes 3 additional doses of the HBV vaccine at 6, 10, and 14 weeks of life. A country’s administrative capacity for tracking an infant’s immunization progress may obstruct the vaccine’s success.^4^^,^^35^ A review that included SSA countries currently administering HepB-BD reported that the vaccine’s documentation is suboptimal across the continent.^4^ A lack of adequate reporting infrastructure (data health information systems)^34^ and failure to comply with vaccine recording by facility staff may explain these findings.

The tracking system may be improved by standardizing all HepB-BD-related immunization-reporting tools, such as immunization cards, registers, and data management systems, to include HepB-BD-specific administration details. Tracking of doses administered also helps to inform supply management of HepB-BD at the facility level.^45^ The use of standardized tools can calibrate and improve data monitoring efforts in LMICs.^34^ Furthermore, monitoring and evaluation systems must be in place to ensure that health workers are appropriately using the developed tracking tools. Training, protocol utilization, and regular assessment of health worker compliance in tracking vaccinations can increase compliance of vaccine documentation across the country.

#### Vaccine Storage and Stock-Outs

Health facility administrators should safeguard access to vaccines by addressing both limited storage space and stock-out determinants. Botswana, The Gambia, Mauritania, Namibia, and Nigeria reported stock-outs and limited vaccine sessions as barriers to timely access and administration of HepB-BD.^4^^,^^33^ Poor communication between the immunization and maternity wards can further hinder vaccine logistics at the facility level.^33^ A final logistical barrier is reaching remote rural villages with vaccines.^37^^,^^52^

Solutions to vaccine stock-outs and limited vaccine sessions include storing the vaccine in existing cold chains, preferably in labor wards, establishing standing orders for the vaccine, and leveraging partnerships with vaccine distributors.^51^

Facilities can use existing storage space or store HepB-BD in the labor ward. Health officials in many SSA countries already recommend that infants receive the Bacillus Calmette-Guerin (BCG) and oral polio vaccines at birth, and both have achieved high rates of uptake.^49^ Storage space in health facilities may therefore already exist, and introducing HepB-BD to the existing cold-chain storage unit may not prove to be a significant disruption.^35^^,^^36^ One study in the Democratic Republic of the Congo leveraged the existing Expanded Programme on Immunization cold-chain infrastructure to provide HepB-BD but uncovered challenges in delivering the vaccine in a timely manner.^51^ A São Tomé and Príncipe study demonstrated that storing the HepB-BD in labor wards is another effective approach to increase HepB-BD rates.^39^ However, this may prove a challenge in some settings where maternity wards are not equipped for vaccine-specific storage requirements. Therefore, effective communication and coordination are needed between immunization clinic staff and maternity staff to ensure that infants receive timely vaccination. National mandates to vaccinate in maternity wards could help facilities and staff to shift birth-dose administration from immunization clinics to maternity wards.

SSA countries that currently administer HepB-BD also reported stock-outs as an implementation barrier. A solution to frequent stock-outs employed in the Western Pacific was establishing standing orders for HepB-BD with the manufacturer.^11^^,^^34^ Vaccine rates increased due to regular shipments of HepB-BD in place of need-based shipments.

SSA countries that currently administer HepB-BD also reported stock-outs as an implementation barrier.

Finally, a public-private partnership with Gavi can facilitate logistics, avoid stock-outs, and lower vaccine costs.^36^ Gavi has overseen the distribution of many vaccines, including HepB-BD, across the globe and can therefore provide empirical knowledge of supply and logistics for recent adopters of HepB-BD. In addition, national vaccination initiatives are advised to partner with private facilities where a substantial portion of deliveries occur to allow private providers to obtain routine HepB-BD free-of-charge, in line with public providers. The Democratic Republic of the Congo provides vaccines for free through public, private, and faith-based facilities to increase immunization reach.^53^ In the Philippines, 20% of deliveries occur in private facilities, so private providers received free vaccines in exchange for data on doses administered.^54^ This approach will increase the accessibility of the HepB-BD vaccine for mother-infant pairs who seek care across different types of facilities.

### Community Level

SSA countries are predominately rural, presenting a unique access-based barrier. In SSA, marginalized people who must travel more than 2 hours to access emergency public hospital facilities make up 29% of the population, of whom 28% are women of childbearing age. In fact, hospital facility access is only widely available in 16 of 48 African countries.^55^ South Sudan presents the most extreme case globally, with 75% of its population living further than a 2-hour walk away from a hospital facility.^55^ Given these statistics, immunization system strengthening cannot happen solely at centralized health facilities. Instead, efforts must also extend to reach mothers and newborns at the community level.

A high proportion of SSA infants are born at home and therefore do not have access to HepB-BD.^35^^,^^56^ Low HepB-BD adherence rates are largely due to the lack of formal outreach programs to vaccinate newborns at home or to refer mother-infant pairs to nearby facilities.^35^^,^^57^ Another determinant is the lack of access to health facilities and trained CHWs to administer the vaccinations. Proposed solutions include improved maternal involvement, CHW involvement, and innovative technologies.

#### Maternal Involvement

The mother-infant pair is integral to the success of any immunization implementation approach. Health officials often consider at-home births inadvisable. However, most home births occur not because the mother refuses to visit a health facility but because she lacks access to a facility.^58^ Therefore, educating mothers about the importance of timely HepB-BD must occur at both the facility and the community levels. Decision makers should consider community-level interventions focused on health behaviors. For instance, cultural barriers may impact the time taken to bring an infant to a facility after delivery. In The Gambia and Nigeria, where at-home birth rates are high, mothers wait 7 days to name their infants before visiting a facility, hindering timely administration of HepB-BD.^34^

While the prevalence of home births may be attributed to cultural, religious, or access-related issues, most women eventually bring their newborns to facilities after birth. Health officials should consider leveraging these visits after a home birth to deliver HepB-BD as soon as possible and educate mothers about the importance of a timely visit.

Additional targeted interventions include raising awareness within communities and building trust by leveraging existing civil society networks, improving understanding, and reminding caregivers of the importance of HepB-BD.^46^ These initiatives allow the most crucial contributors in the implementation formula, the mothers, to have the resources necessary to understand the importance of timely vaccination.

Antenatal care facilities are an essential resource to educate pregnant women about the importance of HepB-BD. More than 90% of SSA women have had at least 1 antenatal care visit; this is an opportune time to teach women about in-facility delivery benefits and the importance of timely HepB-BD.^34^^,^^35^ However, community-based educational initiatives should be simultaneously promoted by establishing a gathering place within a community or using radio or social media campaigns to disseminate vaccination information to expectant mothers. Exposure to information during pregnancy has a positive impact on HBV knowledge and can be achieved by incorporating targeted education programs or improving existing educational material to include targeted HepB-BD information.^59^ A Nigerian study delivered an educational initiative for expectant mothers, providing information on HBV burden in-country and across SSA, vaccine benefits, and the timing of the HepB-BD vaccine.^33^ The study found that immunization education and awareness successfully increased HepB-BD uptake. Information strategies should focus on the vaccine’s safety and be delivered by CHWs to increase HepB-BD uptake.^60^

A final approach overlooked in the HepB-BD-related literature is the use of home-based records as a potential solution to increase immunization coverage at the community level. Home-based records can facilitate mothers’ knowledge and detection of health problems and can encourage continuity of care and completion of the 4 doses of HBV vaccine.^61^

A final approach overlooked in the HepB-BD-related literature is the use of home-based records as a potential solution to increase immunization coverage at the community level.

#### Community Health Worker Involvement

Significant vaccination obstacles exist during at-home births due in large part to fissures in communication between community members and health facilities. CHWs can bridge these fissures, improving vaccine awareness and knowledge in a community.

CHWs can act as intermediaries to provide relevant delivery and vaccination information to mothers, other family members, and health facilities. Notably, CHWs are a critical source of health information for pregnant women and mothers, as well as other community members, in rural locations. CHW-led home visits are vehicles for reaching women who do not have access to antenatal care in rural communities.^46^ Home visits allow skilled birth attendants to educate expectant mothers, identify potential births in the communities, attend the deliveries,^34^ promote institutional deliveries,^12^^,^^62^ and refer the mother-infant pair to nearby facilities for vaccination and other services. In addition, many SSA countries have regular outreach services to provide vaccinations and other services to children living in hard-to-reach villages.^63^ CHWs may even travel to remote communities for scheduled visits and to provide door-to-door immunization for infants.

Given that CHWs are the liaison between communities and a health facility, strong communication ties between CHWs and health facilities are crucial to vaccine uptake. This importance is highlighted by relevant SEA studies, which reported a cost-effective and practical strategy through regular district-level training. CHWs visit health facilities to receive regular training and share learnings with their communities.^46^^,^^52^ A Republic of Kiribati study reported that educating CHWs led to an 18% increase in timely HepB-BD delivery across the island.^52^ CHWs can further advocate for HepB-BD by involving religious and community leaders and by engaging men and other family members to influence community norms and acceptance.^45^ CHWs play a vital role in the success of a vaccine intervention, and incentives to collaborate come either from having a stake in their community's well-being, from per-diems for vaccine interventions,^64^ or encouraging referral of mother-infant pairs to health facilities.^45^ CHWs are often the primary representative of the health care system at the community level. As such, their involvement is essential to ensure community buy-in of HepB-BD.

#### Evidence-based Innovations to Reach Communities

Beyond knowledge barriers, logistical barriers to HepB-BD delivery exist at the community level. HepB-BD coverage in rural communities remains low due to HepB-BD’s cold-chain storage requirements; the vaccine must be kept between 2°C and 8°C.^37^^,^^52^

Increased vaccine coverage is now possible due to scientific (use of out-of-cold-chain [OCC] or controlled-temperature chains) and technological (mHealth) innovations to address geographic barriers to access of HepB-BD.

Various studies have examined the advantages of vaccine storage at ambient temperature at the point of service delivery.^45^ Studies in SEA exploring solutions to reach rural at-home births found that HepB-BD can be kept OCC at a temperature of up to 37°C for 1 month without loss of potency.^4^^,^^37^^,^^65^ While some countries have used OCC approaches to transport other vaccines,^66^ this innovative approach is novel in SSA and has never been incorporated into a model to reach at-home births within a 24-hour window.

WHO recommends taking the OCC approach further by transporting and storing vaccines in stable temperature carriers—controlled-temperature chains (CTCs).^45^ This approach increases coverage, reduces resource wastage, and increases the outreach of HepB-BD.^45^ CTCs require several conditions to maintain the vaccine outside the traditional 2°C to 8°C cold chain, including single removal from the cold chain into temperatures not exceeding 40°C.^45^ Compared with the OCC approach, this innovation halves the cost, reduces the risk of freezing the vaccine, and more effectively protects vaccine quality.^45^ However, unlike traditional OCC technology, CTCs require additional CHW training.^4^ CTC trials conducted in Laos and the Solomon Islands tested the effect of CTCs for HepB-BD transport and storage and reported increases in vaccine uptake by 28%^67^ (Laos) and 150%^68^ (Solomon Islands). Delivering vaccines using CTCs has strong potential to increase vaccine coverage, especially in rural, hard-to-reach communities. CTCs are not currently available as licenses and prequalifications are pending for HepB-BD.^11^^,^^45^

Studies have also reported coupling mHealth aspects with OCC methods for HepB-BD delivery. A Laos study used mobile phones provided to CHWs to track at-home deliveries, real-time stock-outs, and monitor cold-chain temperature following OCC vaccine delivery. The study demonstrated a significant improvement in the proportion of children receiving HepB-BD-related home visits.^64^ Multifaceted innovations could improve HepB-BD coverage in rural villages.

## DISCUSSION

We summarize key determinants of HepB-BD implementation at the 3 levels of policy, facility, and community in SSA (Table 2). We highlight barriers to timely uptake of the vaccine at all levels, as well as evidence-based solutions to these barriers. Any implementation strategy for HepB-BD must consider all 3 levels and must be adapted to the local context. SSA countries should model their implementation strategies on the success stories of HepB-BD introduction in SEA.^52^^,^^59^^,^^60^^,^^64^ There are a few key examples of successful HepB-BD introduction in SSA,^33^^,^^35^^,^^36^ but there is much room for improvement since only 13 of 48 countries have incorporated HepB-BD into their routine vaccination schedules.

Several areas stand out as “low-hanging fruit” for further research and development. Although SSA countries acknowledge the need for buy-in for HepB-BD at the political and facility levels, health officials and researchers continuously understate the mother’s role in HepB-BD implementation approaches. In Nigeria, Botswana, and The Gambia, where HepB-BD policies exist,^39^^,^^64^ the literature focuses predominately on results measured at the facility or national level, rather than at the community level. Notably, significant gaps exist in the literature when addressing the 3 sublevel themes discussed at the community level: maternal involvement, CHW involvement, and evidence-based innovations.

SSA is highly rural, and universal coverage of HepB-BD will prove impossible without reaching at-home births. We suggest future research be geared toward community-level HepB-BD implementation approaches leveraging CHWs and prioritizing mothers. Proposed research includes testing and leveraging evidence-based innovations already identified in SEA^11^^,^^34^^,^^37^^,^^52^ to improve vaccine reach to home births and involving mothers in the implementation process. Despite these potential benefits, existing literature does not address these service end-user approaches in a HepB-BD-specific context. Finally, scientific (OCC vaccines) and technological (mHealth) innovations can drastically improve vaccine uptake in at-home births.

Published literature on HepB-BD immunization implementation in SSA countries is limited, but success stories from diverse settings confirm that HepB-BD is feasible. Synergy must exist between the 3 levels of policy, facility, and community for SSA countries to effectively implement a universal HepB-BD immunization policy. Literature about the mother's role at the community level is strikingly scarce, and efforts to leverage community-level resources have been limited. To achieve successful HepB-BD introduction in African settings, more attention must be paid both by policy makers and researchers to the mother’s and the community's role in vaccine uptake.

## Supplementary Material

GHSP-D-21-00277-Supplement.pdf

## Notes

**Peer Reviewed**

**First published online:** January 21, 2022.
