# Supplementary material for: Implementation Approaches for Introducing and Overcoming Barriers to Hepatitis B Birth-Dose Vaccine in sub-Saharan Africa
Source: Glob Health Sci Pract. 2022 Feb 28;10(1):e2100277. doi: 10.9745/GHSP-D-21-00277 (PMC8885356; doi:10.9745/GHSP-D-21-00277)
Supplement: GHSP-D-21-00277-Supplement.pdf [file GHSP-D-21-00277-Supplement.pdf]

Supplement to: Boisson A, Goel V, Yotebieng M, et al. Implementation approaches for introducing and overcoming barriers to hepatitis B birth-dose vaccine in Africa. *Glob Health Sci Pract*. 2022;10(1):e2100277. https://doi.org/10.9745/GHSP-D-21-00277

Supplement Table. Hepatitis B Birth Dose Vaccine Uptake Themes Discussed in the Empirical Literature (N =39)

Author(s)	SSA Specific	Lack of Political Willingness Advocacy	Lack of Political Willingness Affordability	Effective Recommendations	Knowledge / Training of HWs	Quick Vaccine Administration	Cost Burden	Tracking System	Vaccine Stockouts	Mother Involvement	CHW involvement	Evidence-Based Innovations
Anderson et al., 2018	X		X				X					
Awuku, Yeboah-Afihene, 2018	X	X				X						
Beigi et al., 2014										X		
Boa et al., 2017	X	X	X									
Breakwell et al., 2017								X	X			X
Breakwell et al., 2017	X	X	X	X	X	X		X	X	X	X	X
Centers for Disease Control and Prevention, 2013					X				X		X	
Chang et al., 2019						X						
Dionne-Odom et al., 2018	X	X	X	X	X	X	X		X			X
Giao et al., 2019										X	X	
Ginzberg, Wong, Gish, 2018				X		X						

Supplement to: Boisson A, Goel V, Yotebieng M, et al. Implementation approaches for introducing and overcoming barriers to hepatitis B birth-dose vaccine in Africa. *Glob Health Sci Pract*. 2022;10(1):e2100277. https://doi.org/10.9745/GHSP-D-21-00277

Author(s)	SSA Specific	Lack of Political Willingness Advocacy	Lack of Political Willingness Affordability	Effective Recommendations	Knowledge / Training of HWs	Quick Vaccine Administration	Cost Burden	Tracking System	Vaccine Stockouts	Mother Involvement	CHW involvement	Evidence-Based Innovations
Hagan et al., 2019			X				X	X				
Hambridge et al., 2019	X			X								
Howell, Lemoine, Thursz, 2014	X	X	X	X			X					
Jourdain, Ngo-Giang-Huong, Khamduang, 2019				X			X					
Kolwaite et al., 2016											X	X
Lemoine, Thursz, 2017	X	X	X									
Li et al., 2017					X				X		X	X
Mak et al., 2018												
Miyahara et al., 2016	X					X				X		
Moturi et al., 2018	X					X	X				X	
Nayagam et al., 2016		X	X	X			X					
Nayagam et al., 2016		X	X	X			X					
Nelson, Easterbrook,				X	X						X	X

Supplement to: Boisson A, Goel V, Yotebieng M, et al. Implementation approaches for introducing and overcoming barriers to hepatitis B birth-dose vaccine in Africa. *Glob Health Sci Pract*. 2022;10(1):e2100277. https://doi.org/10.9745/GHSP-D-21-00277

Author(s)	SSA Specific	Lack of Political Willingness Advocacy	Lack of Political Willingness Affordability	Effective Recommendations	Knowledge / Training of HWs	Quick Vaccine Administration	Cost Burden	Tracking System	Vaccine Stockouts	Mother Involvement	CHW involvement	Evidence-Based Innovations
McMahon, 2016												
Nguyen et al., 2019										X		
Okenwa et al., 2019	X	X		X	X	X	X		X	X		
Pham et al., 2018					X	X						
Pham et al., 2019										X		
Reardon et al., 2019	X		X				X					
Scott et al., 2018			X									
Sobel et al., 2011				X	X		X		X			
Spearman, 2018				X								X
Spearmen et al., 2017	X				X			X	X	X	X	
Tamandjou et al., 2017	X	X	X			X				X	X	X
Wiesen et al., 2016					X			X	X	X	X	
Wiesen, Diorditsa, Li, 2016				X								
Woodring et al., 2019						X				X		X

Supplement to: Boisson A, Goel V, Yotebieng M, et al. Implementation approaches for introducing and overcoming barriers to hepatitis B birth-dose vaccine in Africa. *Glob Health Sci Pract*. 2022;10(1):e2100277. https://doi.org/10.9745/GHSP-D-21-00277

Author(s)	SSA Specific	Lack of Political Willingness Advocacy	Lack of Political Willingness Affordability	Effective Recommendations	Knowledge / Training of HWs	Quick Vaccine Administration	Cost Burden	Tracking System	Vaccine Stockouts	Mother Involvement	CHW involvement	Evidence-Based Innovations
							X					
World Health Organization (WHO), 2019				X	X	X		X	X	X	X	X
Xeuatvongsa et al., 2016											X	X
